# Supplementary material for: EnzML: multi-label prediction of enzyme classes using InterPro signatures
Source: BMC Bioinformatics. 2012 Apr 25;13:61. doi: 10.1186/1471-2105-13-61 (PMC3483700; doi:10.1186/1471-2105-13-61)
Supplement: Addtional file 5 — The Java code to format the data files, evaluate and predict. The file enzml_java_code.tar.gz contains the Java code used to format database data to ARFF and XML formats, to execute cross and train-test (jackknife) evaluations and to record evaluation results to database. More information is included in the readme.txt file and the Javadoc files. The code can be used with a MySQL database. To use a different database software, other JDBC drivers might be required. [file 1471-2105-13-61-S5.gz › java_code/enzml2011/doc/uk/ac/ed/inf/taweka/weka/DataSetChecker.html]

DataSetChecker


---


|  |  |  |
| --- | --- | --- |
| **Overview** \| **Package** \| **Class** \| **Use** \| **Tree** \| **Deprecated** \| **Index** \| **Help** | |  |
| **PREV CLASS**   **NEXT CLASS** | **FRAMES**    **NO FRAMES**     **All Classes** |  |
| SUMMARY: NESTED \| FIELD \| CONSTR \| METHOD | DETAIL: FIELD \| CONSTR \| METHOD |  |


---


## uk.ac.ed.inf.taweka.weka Class DataSetChecker

```
java.lang.Object
  uk.ac.ed.inf.taweka.weka.DataSetChecker
```

---

``` public class DataSetChecker extends java.lang.Object ```

Class

**Version:**
:   3 Sep 2010

**Author:**
:   Luna De Ferrari luna.deferrari-at-ed.ac.uk

---

**Constructor Summary**

`DataSetChecker(DataSetManager manager)`


| **Method Summary** | |
| --- | --- |
| `boolean` | `dataMapsAreCorrect(uk.ac.ed.inf.utils.OneToManyMap instanceAttributeMap, uk.ac.ed.inf.utils.OneToManyMap instanceClassMap)`             public boolean initialisationIsCorrect() { return m\_initialisationIsCorrect; } public void setInitialisation(boolean isCorrect) { m\_initialisationIsCorrect = isCorrect; } |
| `ArffProperties` | `getArffProperties()` |
| `DataSetManager` | `getManager()` |
| `java.util.Properties` | `getProperties()` |
| `boolean` | `propertiesAreCorrect()` |

**Methods inherited from class java.lang.Object**

`equals, getClass, hashCode, notify, notifyAll, toString, wait, wait, wait`

**Constructor Detail**

### DataSetChecker

```
public DataSetChecker(DataSetManager manager)
```


**Method Detail**

### dataMapsAreCorrect

```
public boolean dataMapsAreCorrect(uk.ac.ed.inf.utils.OneToManyMap instanceAttributeMap,
                                  uk.ac.ed.inf.utils.OneToManyMap instanceClassMap)
```

:   public boolean initialisationIsCorrect() { return
    m\_initialisationIsCorrect; }
    public void setInitialisation(boolean isCorrect) {
    m\_initialisationIsCorrect = isCorrect;
    }

---


### getArffProperties

```
public ArffProperties getArffProperties()
```

---


### getManager

```
public DataSetManager getManager()
```

---


### getProperties

```
public java.util.Properties getProperties()
```

---


### propertiesAreCorrect

```
public boolean propertiesAreCorrect()
```


---


|  |  |  |
| --- | --- | --- |
| **Overview** \| **Package** \| **Class** \| **Use** \| **Tree** \| **Deprecated** \| **Index** \| **Help** | |  |
| **PREV CLASS**   **NEXT CLASS** | **FRAMES**    **NO FRAMES**     **All Classes** |  |
| SUMMARY: NESTED \| FIELD \| CONSTR \| METHOD | DETAIL: FIELD \| CONSTR \| METHOD |  |


---
